# Chronic Use of a Sensitized Bionic Hand Does Not Remap the Sense of Touch

**DOI:** 10.1016/j.celrep.2020.108539

**Published:** 2020-12-22

**Authors:** Max Ortiz-Catalan, Enzo Mastinu, Charles M. Greenspon, Sliman J. Bensmaia

**Affiliations:** 1 Center for Bionics and Pain Research, Mölndal, Sweden; 2 Department of Electrical Engineering, Chalmers University of Technology, Gothenburg, Sweden; 3 Operational Area 3, Sahlgrenska University Hospital, Mölndal, Sweden; 4 Department of Orthopaedics, Institute of Clinical Sciences, Sahlgrenska Academy, University of Gothenburg, Gothenburg, Sweden; 5 Department of Organismal Biology and Anatomy, University of Chicago, Chicago, IL, USA; 6 Committee on Computational Neuroscience, University of Chicago, Chicago, IL, USA; 7 Grossman Institute of Neuroscience, Quantitative Biology, and Human Behavior, University of Chicago, Chicago, IL, USA; 8 Lead Contact

## Abstract

Electrical stimulation of tactile nerve fibers that innervated an amputated hand results in vivid sensations experienced at a specific location on the phantom hand, a phenomenon that can be leveraged to convey tactile feedback through bionic hands. Ideally, electrically evoked sensations would be experienced on the appropriate part of the hand: touch with the bionic index fingertip, for example, would elicit a sensation experienced on the index fingertip. However, the perceived locations of sensations are determined by the idiosyncratic position of the stimulating electrode in the nerve and thus are difficult to predict or control. This problem could be circumvented if perceived sensations shifted over time to become consistent with the position of the sensor that triggers them. We show that, after long-term use of a neuromusculoskeletal prosthesis that featured a mismatch between the sensor location and the resulting tactile experience, the perceived location of the touch did not change.

## In Brief

Electrical stimulation of tactile nerve fibers can be used to restore touch through a bionic hand. Ortiz-Catalan et al. show that a mismatch between the location of the sensor on the bionic hand and the tactile experience is not resolved after long-term prosthesis use.

## Graphical Abstract

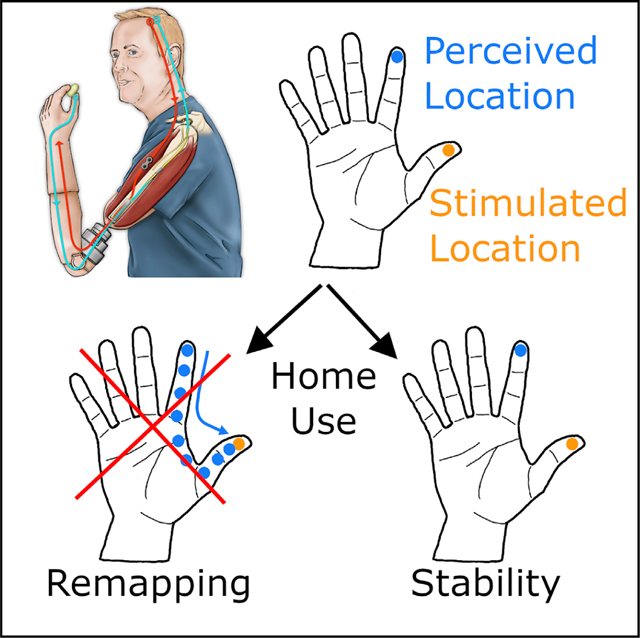

## INTRODUCTION

Manual interactions with objects gives rise to a barrage of neural signals from the skin about the objects themselves (i.e., their size, shape, and texture) and about our interactions with them (i.e., contact timing, force, and location) (Johansson and Flanagan, 2009). Without these tactile signals, dexterous manipulation would be severely impaired, as evidenced by the deficits resulting from digital anesthesia or deafferentation (Johansson et al., 1992). The importance of tactile feedback in manual behavior has spurred the development of strategies to convey tactile signals in bionic hands. One promising approach to sensory restoration is to establish an electrical interface with the residual nerve through chronically implanted electrodes, because microstimulation of the nerve evokes vivid sensations experienced on the phantom hand (Clippinger et al., 1974; Ortiz-Catalan et al., 2014; Tan et al., 2014; George et al., 2019; Petrini et al., 2019).

In principle, the more naturalistic these artificially induced neural signals are, the more intuitive the resulting sensations will be (Saal and Bensmaia, 2015; Valle et al., 2018; George et al., 2019). The most straightforward application of this principle of biomimicry is somatotopic mapping: because stimulation through a given electrode evokes a percept that is localized to a specific patch of skin, connecting a sensor on the corresponding part of the bionic hand to that electrode is likely to convey intuitive information about contact location (Saal and Bensmaia, 2015). For instance, if stimulation through an electrode gives rise to a sensation on the index fingertip, it stands to reason to connect the index fingertip sensor to that electrode: anytime the bionic index fingertip touches an object, the subject will experience a sensation on their fingertip and will thus know where contact was initiated without having to think about it (Dhillon and Horch, 2005).

The problem with the somatotopic mapping strategy is that, in practice, the projection field associated with each electrode, that is, the region of the phantom on which the sensation is experienced when current is delivered through that electrode, is idiosyncratically determined by the location of the electrode on or in the nerve and cannot be prearranged by the implanting surgeon. As a result, a given electrode array may not impinge on some hand regions, one or more fingertips, e.g., where most contact with objects occurs (Christel et al., 1998).

If one cannot control the location of the projection fields, one might hope to relocate them after implantation. Indeed, when the limb region of somatosensory cortex is deafferented through amputation, this deafferented cortex can be activated via touch applied to other body regions (Pons et al., 1991), and amputation of a digit leads to an increase in the neural territory that can be activated through tactile stimulation of adjacent digits (Merzenich et al., 1984). Suturing two digits together leads to a fusing of their cortical representations where the receptive fields of neurons in the somatosensory cortex encompass the two digits as if they were one (Clark et al., 1988). These findings have been interpreted as evidence that body maps may be malleable. The principle underlying reorganization is that it is driven by correlated input: in the case of sutured fingers, both tend to touch the same objects at the same time, and this co-activation leads to the fusing of their cortical representations. One might hope that the chronic pairing of a visual experience of touching one part of the bionic hand, where a touch sensor is located, with a timely tactile sensation to another part of the phantom hand, the projected field of an electrode, will lead to a shift in the perceived location of the sensation, driven by a reorganization of the body map in the brain, itself driven by correlated sensory input.

**Figure 1.**
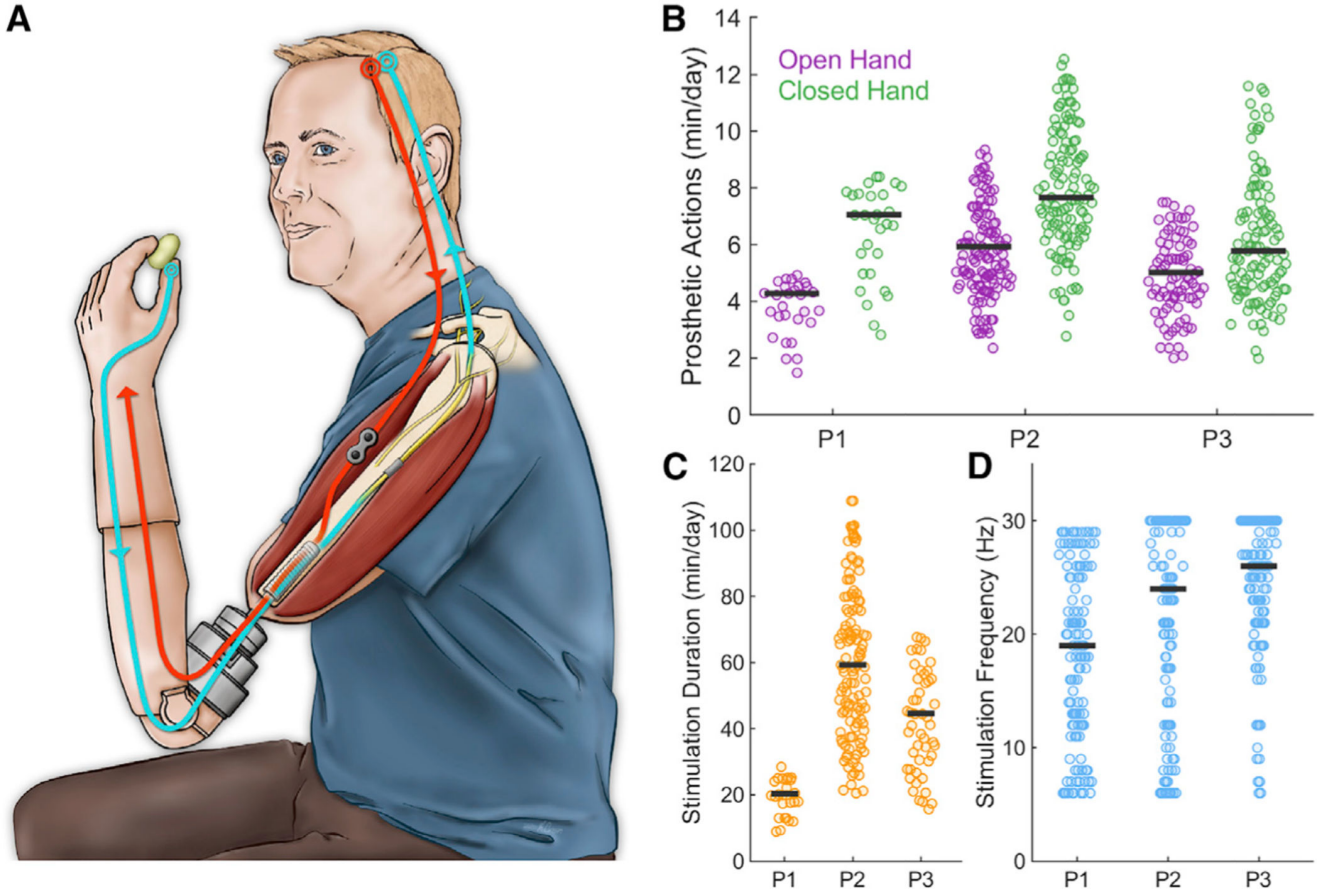
Neuromusculoskeletal Prosthesis Used in Daily Life (A) Participant wearing a neuromusculoskeletalprosthesis connected to his skeleton, nerves, and muscles. Implanted electrodes on muscles and nerves are used for control (red) and sensory feedback (blue), respectively. The interface between internal and external components of the bionic hand is through an osseointegrated implant into the bone. (B) Cumulative time of prosthetic actuation (N_P1_ = 56, N_P2_ = 532, N_P3_ = 222). (C) Cumulative time per day of neurostimulation foreach of the three participants (N_P1_ = 60, N_P2_ = 264, N_P3_ = 102). The prosthesis was worn all the time that the participants were awake during the day. (D) Distribution of stimulation frequencies for the three participants over 100-ms epochs during contact events (N_P1_ = 348861, N_P2_ = 4818708, N_P3_ = 2796206). The horizontal bars represent the median values.

To test this hypothesis, we instrumented three unilateral transhumeral amputees with a neuromusculoskeletal prosthetic arm and hand (Ortiz-Catalan et al., 2014, 2020) (Figure 1A). The hand was controlled via electromyographic signals measured using electrodes implanted on the muscles (Ortiz-Catalan et al., 2012). Tactile feedback was conveyed by electrically stimulating the median or ulnar nerves (Mastinu et al., 2017). Activation of a sensor located on the prosthetic thumb drove electrical stimulation through one electrode contact, dubbed here as the “feedback contact,” implanted around the ulnar (participant 1 [P1]) or median (P2 and P3) nerve. Participants lived with this closed-loop myoelectrically controlled bionic hand and used it to performed activities of daily living for up to 3 years.

## RESULTS AND DISCUSSION

Participants wore the prosthesis every day while awake, except when showering or swimming, based on verbal reports and on-board usage tracking (mean daily usage hours: 18.4, 15.4, and 13.1 h for P1, P2, and P3, respectively). The hand was actuated throughout the day as well, as evidenced by tens of minutes of use for each participant (Figure 1B), implying more than 100 grasping actions per day (assuming each grasping movement lasts an average of a few seconds).

Contact with the prosthetic thumb resulted in electrical stimulation of the nerve for up to 5 s at a time (the duration was capped for safety reasons). The frequency of the electrical stimulation was graded according to the sensor output to modulate the perceived magnitude (Ortiz-Catalan et al., 2020; Dhillon and Horch, 2005; Tan et al., 2014; Graczyk et al., 2016), thereby conveying information about applied pressure. All three participants experienced extensive stimulation each day (mean daily stimulation duration: 20.3, 59.3, and 44.7 min for P1, P2, and P3, respectively; see Figures 1C and 1D).

The prosthetic hand allowed for superior grasping force precision and reliability when compared with conventional surface electrode control (Mastinu et al., 2019). Moreover, the sensory feedback proved beneficial for restoring grasping coordination and assisting corrective actions when grasping under uncertainty, for example, when the weight of the object changed unexpectedly (Mastinu et al., 2020). Additionally, long-term home use of the tactile sensory feedback led to increased sensitivity to changes in electrical stimulation, as evidenced by improved pulse frequency discrimination (Ortiz-Catalan et al., 2020). Furthermore, participants reported greater confidence in their prosthesis control, as well as improved self-image and self-esteem, leading to better social relationships and increased participation in a wider range of activities. Participants also expressed increased embodiment of the bionic limb, claiming that it is “part of my body,” “it is my arm now,” or “I don’t carry it; it is me” (Middleton and Ortiz-Catalan, 2020).

For at least 1 year prior to enabling electrical stimulation of the nerve, participants used their bionic hand without sensory feedback (Ortiz-Catalan et al., 2014). During this period, we tracked the location of the projected field of the different contacts on the cuff electrode. To this end, we periodically delivered a threshold-level microstimulation pulse through one of several contacts (n = 3, 6, and 5 for P1, P2, and P3, respectively), interleaved in random order, and interrogated the subject as to where the sensation was experienced. We also delivered stimulation at the highest intensity used for sensory feedback in daily life (20% over perception threshold at 30 Hz), and the subject reported the extent of the projected field by drawing it on a hand diagram, once approximately halfway through the study and once after it ended. Results from the full mapping are reported elsewhere (Ackerley et al., 2018; Ortiz-Catalan et al., 2020). Here, we present results for the feedback contact, which was paired with the sensor.

**Figure 2.**
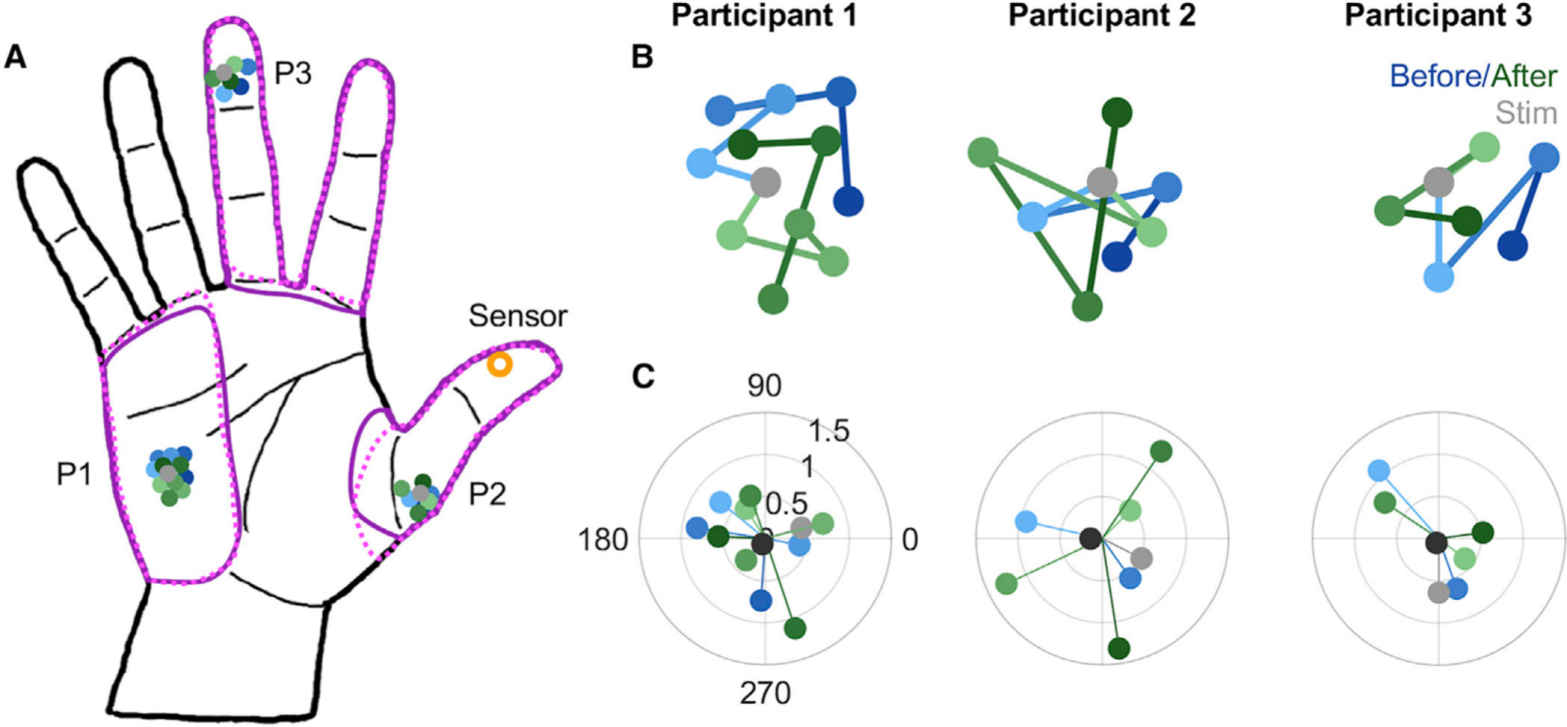
Perceived Location of Electrically Evoked Sensations Does Not Change Despite Chronic Mismatch with Seen Location (A) Location of the projection fields of the feedback electrodes over the course of the study. Gray dots represent the location of the projection fields measured atthreshold on the session when sensory stimulation was activated for home use. Shades of blue and green illustrate the locations before and after this pairing, respectively. Purple outlines denote the extent of the projected field with stimulation at maximum frequency (30 Hz) taken before pairing the feedback electrode (solid) and after the end of the study (dotted). Time periods varied across participants: participant 1 (P1), 27 months before and 28 months after stimulation (12 time points); participant 2 (P2), 10 months and then 8 months (8 time points); and participant 3 (P3), 6 months and then 10 months (7 time points). (B) Sequence of projected fields zoomed in for each participant. (C) Angle and extent of the displacement of the projected field in consecutive measurements. One would expect the angle to be consistent if it was moving systematically toward the sensor, but angles were random (vector strength was not significantly different from what would be observed in the direction, and extent of movement was random from measurement to measurement).

Feedback contacts had projected fields located on the hypothenar (P1), proximal fingerpad of the thumb (P2), and distal fingerpad of the middle finger (P3) (Figure 2A). The location of these projected fields remained consistent over repeated testing during the year preceding the pairing with the sensor (blue hues, Figures 2A and 2B). More importantly, and perhaps surprisingly, the location of the projected field did not change after pairing with the sensor (green hues, Figures 2A and 2B). That is, over the period of over 1 year, every time the participant’s prosthetic thumb contacted an object, they experienced a tactile sensation somewhere else on the hand and the location of that tactile sensation did not change. Periodic testing of the location of the projective field showed that it moved only slightly, typically a millimeter or less, from test to test (typically separated by weeks or months) (Figure 2B). Furthermore, the direction in which the projected field moved was random, as evidenced by vector strengths that were not significantly different from those expected if the direction of movement was uniformly distributed (Figure 2C). Similarly, the extent of the projected fields measured at the highest stimulation intensity was nearly identical after the study as it was before (purple outlines in Figure 2A). These results match participants’ reports, obtained regularly, that the sensations and projected fields remained unchanged throughout the study.

The location of the projection field of the feedback contact was thus remarkably stable, despite the chronic mismatch between the visual experience of contact location and its tactile counterpart. This fixedness is especially surprising given that the prosthesis was used on a daily basis and the sensory feedback was behaviorally relevant (Recanzone et al., 1993). The possibility remains that the visuo-tactile mismatch was not salient enough to promote plasticity. Indeed, the participants may not have looked at their bionic hand frequently enough to experience the visuo-tactile mismatch. Or perhaps contact timing during typical object interactions is consistent enough across bionic fingers that this mismatch was obscured. However, two of the three projection fields were not on fingertips, so this is unlikely. Given these caveats, we cannot exclude the possibility that another approach to remap projection fields, for example, by repeatedly pairing the electrical stimulus with a visual cue at the desired location of the projection field (Rognini et al., 2019), would lead to a remapping of the sensory experience. Furthermore, the projection field and the sensor location can become more tightly aligned through chronic home use of a prosthesis if these are congruent to begin with (Cuberovic et al., 2019; Schofield et al., 2020). For one participant in the present study (P2), however, the projected field at maximum stimulation did overlap with the sensor, but the projected field remained stable nonetheless (Figure 2).

Our results suggest that the visuo-tactile mismatch does not resolve itself when participants perform activities of daily living with the bionic hand, even over an extended period. This finding is consistent with a view that sensory maps are highly stable in adulthood (Makin and Bensmaia, 2017) and cannot be meaningfully modified, even with prolonged exposure.

## STAR★METHODS

### RESOURCE AVAILABILITY

#### Lead Contact

Further information and requests for resources and reagents should be directed to and will be fulfilled by the Lead Contact, Max Ortiz-Catalan (maxo@chalmers.se).

#### Materials Availability

This study did not generate new unique reagents.

#### Data and Code Availability

All original data and code used for analysis and figure generation can be found online at Figshare (https://doi.org/10.6084/m9.figshare.13143236.v1).

### EXPERIMENTAL MODEL AND SUBJECT DETAILS

Three participants with transhumeral amputation implanted with a neuromusculoskeletal arm prosthesis participated in the study. Details on the participants medical background is provided in reference (Ortiz-Catalan et al., 2020). Participant 1 (P1) was implanted in 2013 (Ortiz-Catalan et al., 2014), Participants 2 and 3 (P2 and P3) in 2017. All participants were males, ages 46, 45, and 43 years old, respectively. P2 and P3 underwent a targeted muscle reinnervation (TMR) surgical procedure aimed at providing intuitive myoelectric signals for hand opening and closing (Kuiken et al., 2009). The study was approved by the Swedish regional ethical committee in Gothenburg (Dnr: 769–12) and all participants provided written informed consent.

### METHOD DETAILS

#### Neuromusculoskeletal arm prosthesis

The neuromusculoskeletal interface (e-OPRA, Integrum AB, Sweden) consists of 1) an osseointegrated percutaneous titanium implant for direct skeletal attachment of the artificial limb, 2) feedthrough connectors embedded in the osseointegrated implant to allow the artificial limb to communicate with implanted electrodes, and 3) implanted electrodes in nerves and muscles with up to 16 electrode contacts (Ortiz-Catalan et al., 2020). Epimysial electrodes were sutured on both naturally innervated and surgically reinnervated muscles, and spiral cuff electrodes were wrapped around the ulnar nerve for P1, and the ulnar and median nerves for P2 and P3 (Ortiz-Catalan et al., 2012). A custom-designed embedded electronic system placed at the interface between the neuromusculoskeletal interface and the prosthesis was used for signal processing, control, and neurostimulation (Mastinu et al., 2017).

#### Prosthetic setup and control

The prosthetic setup for all participants consisted of a myoelectric hand (SensorHand, Ottobock, Germany), elbow (ErgoArm, Ottobock, Germany) and the artificial limb controller (ALC), a custom-designed embedded system for closed-loop prosthetic control that serves the dual purpose of recording EMG to control prosthesis movement and providing sensory feedback via neural stimulation (Mastinu et al., 2017). The prosthesis was self-contained and did not require external batteries, processing, or stimulation equipment. Myoelectric signals from the epimysial electrodes were sampled at 500 Hz, high-pass filtered at 20 Hz, low-pass filtered at 250 Hz, and notch-filtered at 50 Hz. The prosthetic hand was commanded using direct control (also known as one-for-one control), where the mean absolute value of an EMG channel (over a 100-ms time window) was proportionally mapped to the actuation speed. The thresholds for direct control were customized for each participant to provide optimal control of the terminal device.

#### Sensory feedback for home-use

Participants were provided with tactile sensory feedback for home-use in January 2017 (P1) and September 2018 (P2 and P3). Electrical stimulation of the residual nerves via cuff electrodes depended to the output of three sensors located on the prosthetic thumb. The average readout of the force sensors was linearly mapped to the pulse frequency within the range from 5 Hz to 30 Hz (Günter et al., 2019). Stimulation stopped when the sensors were no longer in contact with an object or after 5 s, whichever happened first.

Stimulation pulses were cathodic-first, rectangular, bipolar (50 μs inter-pulse delay), asymmetric (10:1), charge-balanced, and current-controlled. Only one contact of the cuff electrode per participant was used for home-use stimulation, prioritizing ones that required the least charge to elicit perception, that is, the ones yielding the lowest detection threshold. Perceptual threshold was measured by delivering single pulses at different amplitudes and widths and having subjects report whether or not they felt the stimulus. Charge was gradually increased until the subject reported a tactile percept. This procedure was repeated on all the electrodes and the pulse width yielding the lowest charge threshold was identified for each electrode

### QUANTIFICATION AND STATISTICAL ANALYSIS

All analyses were performed using MATLAB 2019b (Mathworks, MA, USA). Three participants contributed to the study and numbers of events recorded for each is reported in the figure legends.

The projected field – the location at which a tactile percept was experienced – was reported by the participant by marking it on an image such as that shown in Figure 2A in the main text. For most measurements, threshold level stimulation was delivered, which resulted in highly localized tactile percepts, reported by the participants to feel like “being touched with the tip of a pen.” On a few occasions (once before the feedback lead was connected, once approximately halfway through the take home trial with sensory feedback, and once at the end of it), we also stimulated at the maximum frequency (30 Hz) and had subjects draw the extent of the projected field on a tablet with their intact hand. Electrical stimulation was never reported as painful.

In addition to the systematic reports of projected field location, subjects were interviewed by phone every week during the first month after sensory feedback was enabled, and then every month for a year, to inquire on any changes in projected field.

#### Characterizing the progression of projected fields

To characterize the progression of the projected fields over time, we first plotted their trajectory in two dimensions (Figure 2B). We then produced a polar plot of the displacement direction and extent between each measurement (Figure 2C). We could then assess whether the projected fields tended to move in any one direction. To this end, we computed the vector strength (Mardia, 1975), given by:

$$vs=\frac{\sqrt{{\left[\sum_{j}d_{j}\sin\left(\theta_{j}\right)\right]}^{2}+{\left[\sum_{j}d_{j}\cos\left(\theta_{j}\right)\right]}^{2}}}{\sum_{j}d_{j}}$$

where *d*_*j*_ is the distance over which and *θ*_*j*_ is the direction in which the projection field moved from one measurement to the next. We then characterized using a Monte Carlo simulation the distribution of vector strengths that would be obtained if the direction was randomized from step to step (by sampling them from a uniform distribution from 0 to 2π), matching the number of steps. Repeating this sampling 10,000 times, we computed the proportion of times the measured vector strength was larger than what would expected by chance, the equivalent of a p value for each measured vector strength.

## Figures and Tables

**Table:** KEY RESOURCES TABLE

REAGENT or RESOURCE	SOURCE	IDENTIFIER
Deposited Data		

Ethical Application	Swedish regional ethical committee in Gothenburg	Dnr: 769–12
Projected fields of perception	This paper (https://doi.org/10.6084/m9.figshare.13143236.v1)	N/A
Cumulative times of prosthetic home usage	This paper (https://doi.org/10.6084/m9.figshare.13143236.v1)	N/A

Software and Algorithms		

ALC control and stimulation algorithms	Mastinu et al., 2017; Integrum AB, Sweden	N/A
Statistical analysis for Figure 2	This paper (https://doi.org/10.6084/m9.figshare.13143236.v1)	N/A

Other		

e-OPRA neuromusculoskeletal interface	Ortiz-Catalan et al., 2014; Integrum AB, Sweden	N/A
Artificial Limb Controller (ALC)	Mastinu et al., 2017; Integrum AB, Sweden	N/A

## Highlights

- Electrical stimulation of tactile nerve fibers evokes vivid touch sensations
- Nerve stimulation can be used to convey tactile feedback through bionic hands
- Mismatch between sensor location and felt location does not resolve with time
